# Minimal Kidney Disease Phenotype in *Shroom3* Heterozygous Null Mice

**DOI:** 10.1177/20543581231165716

**Published:** 2023-06-07

**Authors:** Allison Lawlor, Kristina Cunanan, Joanna Cunanan, Amy Paul, Hadiseh Khalili, Doyun Ko, Ahsan Khan, Robert Gros, Thomas Drysdale, Darren Bridgewater

**Affiliations:** 1 Department of Pathology and Molecular Medicine, Faculty of Health Sciences, McMaster University, Hamilton, Ontario, Canada; 2 Toronto General Hospital Research Institute, University Health Network, Ontario, Canada; 3 Department of Physiology and Pharmacology, Schulich School of Medicine & Dentistry, Western University, London, Ontario, Canada

**Keywords:** Shroom3, chronic kidney disease (CKD), renal tubular epithelium, kidney function, apical-basolateral cell orientation

## Abstract

**Background:**

Shroom family member 3 (SHROOM3) encodes an actin-associated protein that regulates epithelial morphology during development. Several genome-wide association studies (GWAS) have identified genetic variances primarily in the 5’ region of SHROOM3, associated with chronic kidney disease (CKD) and poor transplant outcomes. These genetic variants are associated with alterations in Shroom3 expression.

**Objective:**

Characterize the phenotypic abnormalities associated with reduced *Shroom3* expression in postnatal day 3-, 1-month and 3-month-old mice.

**Methods:**

The Shroom3 protein expression pattern was determined by immunofluorescence. We generated *Shroom3* heterozygous null mice (*Shroom3*^Gt/+^) and performed comparative analyses with *wild type* littermates based on somatic and kidney growth, gross renal anatomy, renal histology, renal function at postnatal day 3, 1 month, and 3 months.

**Results:**

The Shroom3 protein expression localized to the apical regions of medullary and cortical tubular epithelium in postnatal *wild type* kidneys. Co-immunofluorescence studies confirmed protein expression localized to the apical side of the tubular epithelium in proximal convoluted tubules, distal convoluted tubules, and collecting ducts. While *Shroom3* heterozygous null mice exhibited reduced Shroom3 protein expression, no differences in somatic and kidney growth were observed when compared to *wild type* mice. Although, rare cases of unilateral hypoplasia of the right kidney were observed at postnatal 1 month in *Shroom3* heterozygotes. Yet renal histological analysis did not reveal any overt abnormalities in overall kidney structure or in glomerular and tubular organization in *Shroom3* heterozygous null mice when compared to *wild type* mice. Analysis of the apical-basolateral orientation of the tubule epithelium demonstrated alterations in the proximal convoluted tubules and modest disorganization in the distal convoluted tubules at 3 months in *Shroom3* heterozygotes. Additionally, these modest abnormalities were not accompanied by tubular injury or physiological defects in renal and cardiovascular function.

**Conclusion:**

Taken together, our results describe a mild kidney disease phenotype in adult *Shroom3* heterozygous null mice, suggesting that Shroom3 expression and function may be required for proper structure and maintenance of the various tubular epithelial parenchyma of the kidney.

## Abrege

**Contexte:**

Le gène SHROOM3 (membre 3 de la famille Shroom) code pour une protéine associée à l’actine qui régule la morphologie épithéliale pendant le développement. Plusieurs études d’association à l’échelle du génome (GWAS — *Genome-wide association studies*) ont identifié des variations génétiques, principalement dans la région 5’ du gène SHROOM3, associées à l’insuffisance rénale chronique (IRC) et à de mauvais résultats de transplantation. Ces variations génétiques sont associées à des altérations dans l’expression de (Shroom3).

**Objectif:**

Caractériser les anomalies phénotypiques associées à une diminution de l’expression de Shroom3 chez des souris à l’âge postnatal de 3 jours, 1 mois et 3 mois.

**Méthodologie:**

Le profil d’expression des protéines Shroom3 a été déterminé par immunofluorescence. Nous avons généré des souris hétérozygotes *Shroom3* (*Shroom3*^Gt/+^) et procédé à des analyses comparatives avec des congénères de *type sauvage* en ce qui concerne la croissance somatique et rénale, l’anatomie rénale, l’histologie rénale et la fonction rénale à l’âge postnatal de 3 jours, 1 mois et 3 mois.

**Résultats:**

L’expression de la protéine Shroom3 est localisée dans les régions apicales de l’épithélium tubulaire médullaire et cortical des reins des souris de *type sauvage* après la naissance. Des études de co-immunofluorescence ont confirmé l’expression des protéines localisée sur le côté apical de l’épithélium tubulaire dans les tubules contournés proximaux, les tubules contournés distaux et les tubes collecteurs. Les souris hétérozygotes *Shroom3* ont présenté une expression réduite de la protéine Shroom3, mais aucune différence dans la croissance somatique et rénale n’a été observée par rapport aux souris de *type sauvage.* Cependant, de rares cas d’hypoplasie unilatérale du rein droit ont été observés à l’âge postnatal de 1 mois chez les souris hétérozygotes *Shroom3*. L’analyze histologique rénale n’a révélé aucune anomalie manifeste dans la structure globale des reins ou dans l’organization des glomérules et des tubules chez les souris hétérozygotes *Shroom3* par rapport aux souris de *type sauvage*. L’analyze de l’orientation apicale-basolatérale de l’épithélium tubulaire a montré des altérations dans les tubules contournés proximaux et une légère désorganisation dans les tubules contournés distaux à l’âge de 3 mois chez les souris hétérozygotes *Shroom3*. En outre, ces légères anomalies n’étaient pas accompagnées d’une lésion tubulaire ou d’anomalies physiologiques dans la fonction rénale et cardiovasculaire.

**Conclusion:**

Pris dans leur ensemble, nos résultats décrivent un phénotype d’insuffisance rénale légère chez les souris hétérozygotes *Shroom3* adultes, ce qui suggère que l’expression et la fonction de la protéine Shroom3 peuvent être nécessaires pour la structure et le maintien appropriés des différents parenchymes épithéliaux tubulaires du rein.

## Introduction

Genome-wide association studies (GWAS) have identified significant associations between genetic variants in the SHROOM3 gene, and the risk for chronic kidney disease (CKD).^1-3^ There are 4 Shroom family member proteins implicated in various morphological processes, but Shroom3 is considered unique as it is the only member capable of initiating apical constriction.^4^ Traditionally, Shroom3 modulates the direct recruitment and subcellular organization of F-actin and Rho-kinase to induce epithelial cell shape changes through apical constriction.^5-7^ This role in actomyosin regulation is important for epithelial morphogenesis of essential organs ranging from the gut, lens, neural tube, heart, and in our focus, the kidneys.^8-10^ Moreover, Shroom3 is implicated in various developmentally important pathways including apicobasal cell elongation, planar cell polarity signaling, and rosette formation among others.^8,11,12^

Despite the strong associations of Shroom3 with kidney disease, its role in renal epithelial maintenance, repair, and development is not well established. To outline potential roles for *Shroom3* in the kidneys, functional genetic variation studies have begun investigating the impact of altered *Shroom3* expression on kidney development, function, and disease. These studies have found significant associations between genetic variants in *SHROOM3* and CKD, baseline estimated glomerular filtration rate (eGFR), urinary albumin-to-creatinine ratio (uACR), and blood urea nitrogen.^3,13-15^ Studies investigating mouse models with varying *Shroom3* expression levels support these findings and attempt to uncover the cause of these significant associations to further understand the role of Shroom3 in the kidneys. For example, during kidney development, it was established that Shroom3 plays a role in regulating glomerular development and is required for normal podocyte architecture and function. This has been established in studies that characterized the embryonic phenotype of *Shroom3* homozygous null mice showing glomerular abnormalities and disruptions in podocyte cytoarchitecture during embryogenesis and a reduction in glomerular number at embryonic (E) day 18.5.^16^ However, the investigation behind the phenotype of *Shroom3* homozygous null mice was not extended to the postnatal adult kidney as these mice were embryonically lethal due to neural tube morphogenesis failure resulting in exencephaly.^17^ Therefore, studies analyze *Shroom3* heterozygous null mice postnatally to investigate the effects of reduced *Shroom3* expression in adult mice kidneys. The postnatal *Shroom3* heterozygous null mice phenotype at 1-year-old exhibits focal segmental glomerulosclerosis with thickened Bowman’s capsules, albuminuria, and podocyte foot process effacement.^16,18^ Several other studies analyzing *Shroom3* genomic variants have also demonstrated podocyte cytoskeletal abnormalities leading to albuminuria and CKD.^18-20^ Furthermore, in a model of acute kidney injury resulting from ischemia reperfusion, 3-month old *Shroom3* heterozygous null mice exhibited poor tissue recovery as a result of alterations in epithelial repair and redifferentiation after kidney injury.^21^ Studies have also demonstrated poor renal allograft function and interstitial fibrosis from donor kidneys that exhibit *Shroom3* overexpression resulting from a single nucleotide polymorphism risk allele at intronic rs17319721.^22^

In this study, we investigated *Shroom3* heterozygous null mice at postnatal day 3, postnatal day 30, and postnatal day 90 to determine the functional and phenotypic consequences of reduced *Shroom3* expression. We show that reduced *Shroom3* expression causes altered apical-basolateral epithelial polarity in select adult nephron segments and sometimes results in hypoplastic unilateral kidneys. Altogether, these studies demonstrate that *Shroom3* heterozygous null mice exhibit a mild kidney disease phenotype at 3 months that does not result in overt kidney damage or decreased kidney function.

## Methods

### Mouse Models

*Shroom3* heterozygous mutant mice Shroom3Gt(ROSA)53Sor/J (or *Shroom3*^Gt/+^) were gifted from Dr. Thomas Drysdale at the University of Western Ontario and originally generated by Hildebrand and Soriano.^17^ These mice contain a gene trap cassette, SAβgalCrepA, that contains an adenovirus splice acceptor (SA), a bifunctional gene encoding a fusion between β-galactosidase (β-gal), Cre recombinase, and an MC1 polyadenylation (pA) sequence.^23^ This cassette is inserted between exons 3 and 4 of the *Shroom3* gene, resulting in the elimination of the short isoform and truncation of the long isoform of *Shroom3*.^17^ Due to the embryonic lethality of homozygous *Shroom3* mutants, the line is maintained using *Shroom3* gene trap mice that only carry one copy of the gene trap allele (*Shroom3*^Gt/+^).

### Tissue Processing and Histology

Whole kidneys from postnatal day 3, 1 month, and 3-month-old *Shroom3*^Gt/+^ and CD1 *wild type* mice were resected and imaged using a Leica EZ4 dissecting microscope and imaged using LAS software. All kidneys were fixed in 4% paraformaldehyde at 4^°^C for 48 hours and paraffin embedded. Kidneys were sectioned to 5 µm on a Leica microtome, mounted on Superfrost plus microscope slides (VWR, Mississauga, Ontario), and incubated overnight at 37^o^C. Tissue sections were either stained with hematoxylin and eosin (H&E) (Sigma Aldrich, Oakville, Ontario) or utilized for immunofluorescence. All histology images were acquired using the Olympus BX80 light microscope and Cell-Sens image acquisition software. For immunofluorescence and immunohistochemistry, kidney sections were deparaffinized and re-hydrated using xylene and graded ethanol washes (100%, 95%, 70%, and 50%) then washed in phosphate buffered saline (PBS). Antigen retrieval was performed for 5 minutes in a pressure cooker with 11.4 mmol/L sodium citrate buffer solution (pH 6.0). For immunofluorescence, samples were blocked with 7.5% normal goat serum and 4.5% bovine serum albumin at room temperature for 1 hour, followed by incubation with primary antibodies **Shroom3** (gifted from Timothy Plageman, 1:200), **SGLT2** (Santa Cruz, 1:300, Dallas, Texas), **SLC12A3** (ThermoFisher Scientific, 1:200, Burlington, Ontario), **Aquaporin-2** (Santa Cruz Biotechnology, 1:200, Dallas, Texas), and **CD44** (Santa Cruz Biotechnology, 1:200, Dallas, Texas) overnight at 4^°^C. Primary antibodies were diluted in an incubation buffer containing 1% PBS, 3% bovine serum albumin, 5% normal goat serum, and 0.3% Tween20. Tissue samples were washed 3 times in PBS and incubated with secondary antibodies Alexa Fluor 488 anti-rabbit (Life Technologies, 1:1000, Milton, Ontario) and DyLight 594 anti-mouse (Invitrogen, 1:1000, Burlington, Ontario) in incubation buffer at room temperature for 1 hour, washed in PBS, counterstained with DAPI (Sigma Aldrich), washed in PBS again, then fixed using Fluoromount mounting medium (Sigma Aldrich, Oakville, Ontario). Immunohistochemistry was performed using the Vectastain Elite avidin-biotin complex kit (Vector Laboratories, Newark, California). After antigen retrieval, slides were immersed in 3% hydrogen peroxide at room temperature for 30 minutes followed by blocking with 5% normal goat serum in PBS with Tween20 (PBST) at room temperature for 30 minutes. Avidin and biotin blocking reagents (Vector Laboratories, Newark, California) were then applied as per manufacturer protocols. **KIM-1** (Novus Biologicals, 1:00, Littleton, Colorado) primary antibody was used diluted in PBS, and incubated overnight at 4^°^C. Samples were then washed with PBS and incubated with biotinylated goat anti-rabbit secondary antibodies in PBST (Vector Laboratories; 1:200, Newark, California) at room temperature for 45 minutes. Avidin-Biotin Complex (ABC) reagent (Vector Laboratories, Newark, California) was applied on the samples followed by colorimetric visualization using diaminobenzidine (DAB; Vector Laboratories, Newark, California) as per manufacturer protocols. Slides were counterstained with hematoxylin (Sigma Aldrich, Oakville, Ontario), dehydrated using graded ethanol washes (50%, 75%, 95%, and 100%) and xylene washes, followed by mounting using Vectamount mounting medium (Vector Laboratories, Newark, California). All images were acquired using the Olympus BX80 light and fluorescence microscope, and CellSens image acquisition software.

### Blood Pressure Measurements (Tail-Cuff)

Blood pressures of conscious mice were measured using a CODA 6-Channel High Throughput Non-Invasive Blood Pressure system (CODA-HT6, Kent Scientific, Torrington, Connecticut) according to the manufacturer’s instructions. The apparatus applied the tail-cuff method to determine blood pressure. Mice were placed into holders that restrained movement; the head was secured with a nose cone while the tail extended out of the rear end of the holder. The holders were kept on a warming platform to maintain the mice at a minimum temperature of 32°C for the duration of the experiment. Two cuffs were placed around the tail: an occlusion cuff which impedes blood flow by inflation, and a Volume Pressure Recording (VPR) cuff which measures the swelling of the tail that occurs as a result of returning blood flow upon deflation of the occlusion cuff. The VPR sensor measures and outputs 6 different physiological readings: systolic and diastolic blood pressure, mean arterial pressure, heart rate, tail blood flow, and tail blood volume. To condition the mice and minimize the level of stress caused by the tail-cuff apparatus, mice were trained daily for 2 to 3 weeks prior to taking the basal blood pressure measurement. Blood pressures were monitored over 2 sets of 15 cycles each, with 5 additional acclimatization cycles at the start of the first set; measurements were taken at the same time each day.

### Body Weight and Kidney Weight Measurements

Mice were weighed at postnatal day 3, 1 month, and 3 months, then euthanized by carbon dioxide. The kidneys were extracted, trimmed of excess tissue, and washed in ice-cold PBS. Kidneys were blotted dry and weighed on the same scale used to measure body weight. Kidney weights were only obtained at postnatal 1 month and 3 months as the postnatal day 3 kidneys were too small to obtain accurate weight measurements.

### Urinalysis

Urine was collected on various days, and collection always occurred at 9:00 AM on each day. To induce urination, the mice were put on a cold glass plate where they were scruffed and pressure was directly applied to the bladder area. The urine was transferred to an Eppendorf tube and then stored at -80°C until analysis. Urine was analyzed for urinary protein and creatinine levels using ChemStrip 10 dip sticks (Roche, Pickering, Ontario) (Western University Hospital, London, Ontario). Urine was also analyzed for the presence of glucose using BioStrip reagent strips (Innovatek, Vancouver, British Columbia).

### Statistical Analysis

Two-tailed *t*-tests were performed using GraphPad Prism (v8.1.2, San Diego, California) to compare levels in *wild type* and *Shroom3*^Gt/+^ mice, *P*-values of <.05 were considered statistically significant and data were reported as mean ± SEM.

## Results

### Results 1: Normal Shroom3 Protein Expression in the Tubular Epithelium

**Figure 1.**
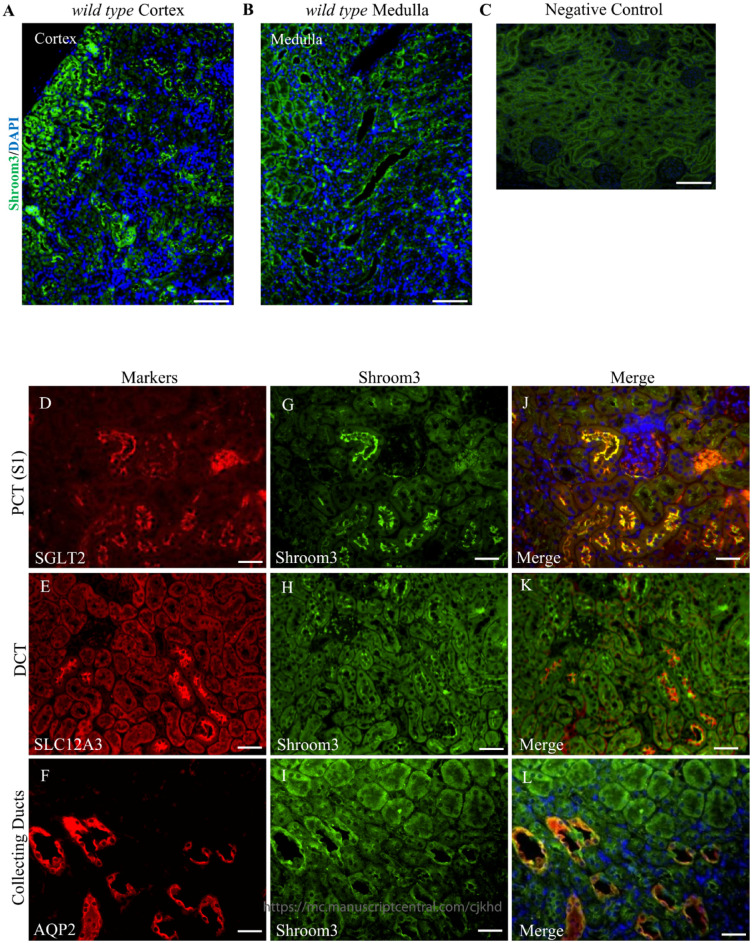
Shroom3 is expressed in the kidney tubular epithelium. **(A and B)** Immunofluorescence staining of adult 3-month-old *wild type* kidneys represent Shroom3 protein expression in the proximal convoluted tubules, distal convoluted tubules, and collecting ducts. Shroom3 expression levels is observed in the cortical tubules and medulla. (**C**) Immunofluorescence staining of adult 3-month-old *wild type* kidneys demonstrate no specific background staining of the anti-rabbit secondary antibody in the absence of the Shroom3 primary antibody. **(D-L)** Co-immunofluorescence staining of Shroom3 with known markers of the proximal convoluted tubules (SGLT2), distal convoluted tubules (SLC12A3), and collecting ducts (AQP2) confirm expression in these tubules. (**J-L**) Shroom3 is apically expressed in all the tubular epithelium analyzed. Scale bars = 50 µm (A-C). Scale bars = 25 µm (D-L).

Our previous studies characterized the expression and function of Shroom3 in both embryonic and adult mouse kidneys. These studies revealed that Shroom3 is expressed in the glomeruli and is responsible for maintaining normal podocyte cytoarchitecture and function by modulating the cellular actomyosin network.^16^ In this study, we built on these previous findings by expanding the expression pattern characterization of Shroom3 to the kidney tubular epithelium. Immunofluorescence in adult 3-month-old mice revealed apical Shroom3 expression in the cortical and medullary tubules, with higher levels of expression seen in the cortical tubules (**Figure 1A and B**). To distinguish which tubular epithelial segments express Shroom3, we performed co-immunofluorescence using known markers of the proximal convoluted tubule (sodium glucose transporter–Sglt2) (**Figure 1D**), distal convoluted tubule (solute carrier Family 12-member 3- Slc12a3) (**Figure 1E**), and collecting duct (aquaporin 2- Aqp2) (**Figure 1F**). These results indicate that Shroom3 is apically expressed in each of the studied tubule segments (**Figure 1G-L**) with the most robust expression observed in the S1 segment of the proximal convoluted tubules (**Figure 1G**). Taken together, these studies describe the normal characterization of Shroom3 in the tubule epithelium demonstrating that Shroom3 is most highly expressed in the cortical tubules, particularly the S1 segment of the proximal convoluted tubules.

### Results 2: Shroom3^Gt/+^ Mice Do Not Show Gross and Histological Abnormalities

**Figure 2.**
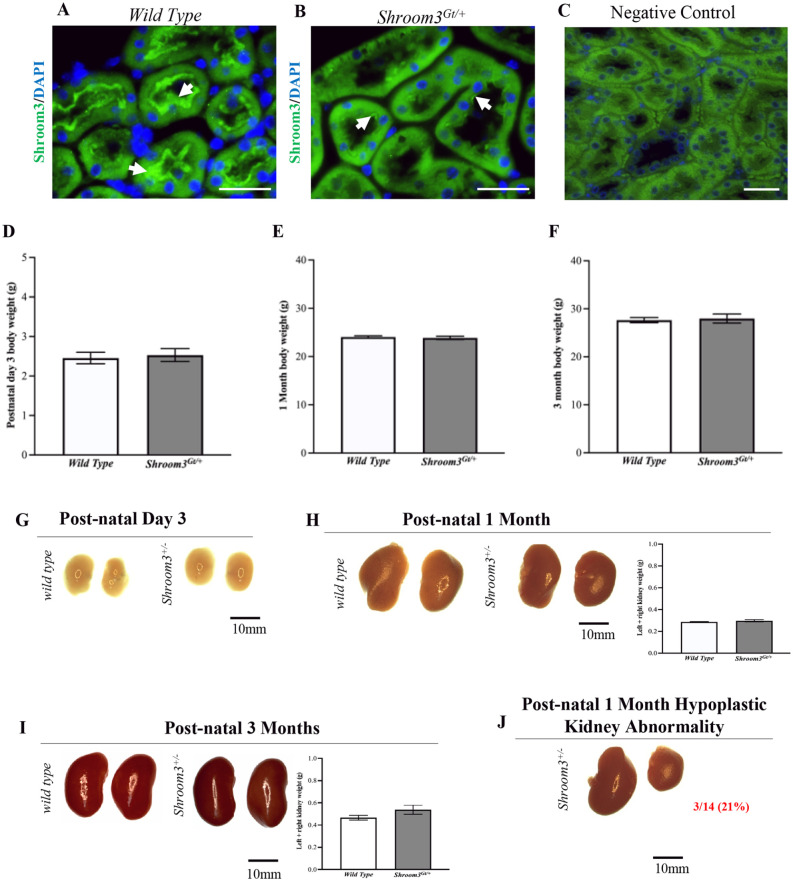
Heterozygous Shroom3 mutant kidney expression pattern. (**A and B**) Immunofluorescence staining in *wild type* and *Shroom3*^Gt/+^mice demonstrates apical Shroom3 expression. Although reduced Shroom3 expression levels are observed in *Shroom3*^Gt/+^mice expression remains apically localized. (**C**) Immunofluorescence staining of adult 3-month-old *wild type* kidneys demonstrates no specific background staining of the anti-rabbit secondary antibody in the absence of the Shroom3 primary antibody. (**D-F**) Total body weight at postnatal day 3, 1 month, and 3 months revealed no statistically significant differences indicating no abnormalities in somatic growth in *Shroom3*^Gt/+^mice. (**G-I**) Kidney resection of *wild type* and *Shroom3*^Gt/+^mice at postnatal day 3, 1 month, and 3 months. The kidney gross anatomy is similar in *wild type* and *Shroom3*^Gt/+^mice. (**J**) Although, there were rare occurrences of *Shroom3*^Gt/+^mice showing hypoplastic unilateral kidneys predominantly observed in the right kidney of heterozygous mice at 1 month. (**H and I**) Combined kidney weight analysis at postnatal 1 month and 3 months show no statistically significant differences. Scale bars = 10µm (A-B). Scale bars = 25µm (C).

**Figure 3.**
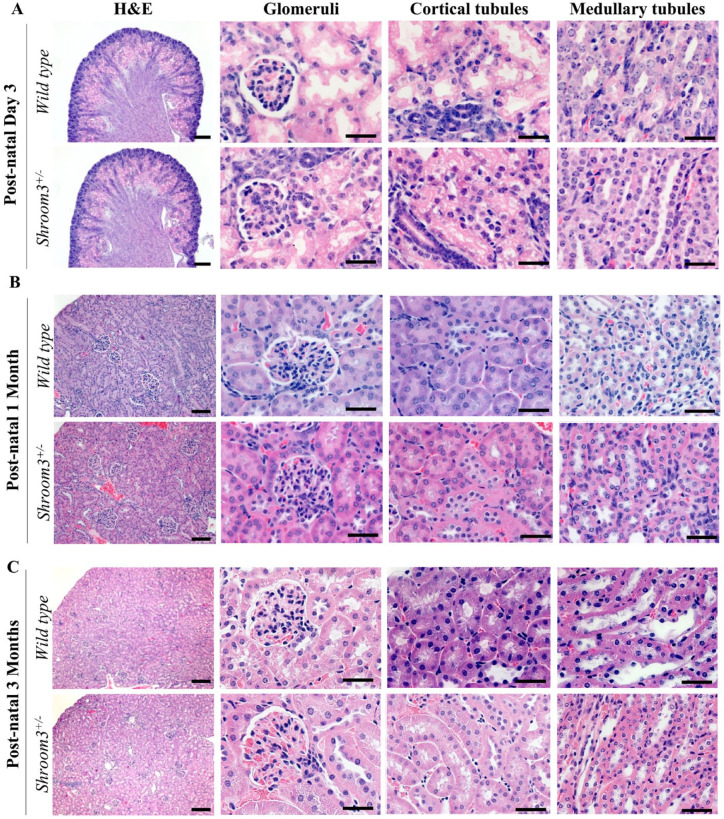
*Shroom3*^Gt/+^mice do not exhibit overt histological abnormalities. (**A-C**) Hematoxylin and eosin staining of postnatal day 3, 1 month, and 3 months *wild type* and *Shroom3*^Gt/+^whole kidney, glomerulus, cortical tubule, and medullary tubule sections, revealed no overt significant differences. Scale bars = 200µm (H&E: A-C low power), Scale bars = 25µm (A-C Glomeruli, Cortical tubule, Medullary tubules: high power).

We performed a series of experiments to establish an overall characterization of the *Shroom3*^Gt/+^mice. In comparing the Shroom3 expression pattern between *wild type* and *Shroom3*^Gt/+^mice, it was confirmed that the heterozygous null mice exhibited decreased Shroom3 expression levels by immunofluorescence. However, while there is reduced protein expression of Shroom3 in the *Shroom3*^Gt/+^mice, the expression pattern remains apically localized in both the *wild type* and *Shroom3*^Gt/+^mice (**Figure 2A and B**). The *Shroom3*^Gt/+^mice were born with expected Mendelian ratios and survived equally as well as *wild type* mice, indicating no embryonic or perinatal lethality. Next, we evaluated the total body weight at postnatal day 3, 1 month, and 3 months. This revealed no differences between *wild type* and *Shroom3*^Gt/+^mice, demonstrating Shroom3 does not have any effects on somatic growth up to 3 months (**Figure 2D-F**). To determine whether abnormalities presented at the whole kidney level, we resected kidneys from both *wild type* and *Shroom3*^Gt/+^ mice. There were no statistically significant differences (*P* > .05) in the combined kidney weights between the 2 strains at postnatal 1 and 3 months (postnatal day 3 kidneys did not exhibit reliable/repeatable results due to their small size) (**Figure 2H and I**). There were no observable differences in the gross morphology of all organ systems nor in the kidney gross anatomy for most mice at postnatal day 3, 1 month, and 3 months (**Figure 2G-I**). However, we observed a few postnatal day 3 mice with an observable smaller contralateral kidney, an observation exaggerated at postnatal 1 month. We noticed 3/14 *Shroom3*^Gt/+^ mice exhibited a hypoplastic unilateral kidney. This abnormality was predominantly observed in the right kidneys of female *Shroom3*^Gt/+^mice and was never observed in *wild type* mice (**Figure 2J**). Despite our previous studies showing observable histological differences in embryonic *Shroom3*^Gt/+^ mice, in this study, the *Shroom3*^Gt/+^mice were indistinguishable from the *wild type* mice at all 3 studied ages.^16^ There were no marked abnormalities in the glomerular, tubular, and overall renal parenchyma organization, even in the rare occurrence of the hypoplastic unilateral kidney (**Figure 3A-C**). Altogether, these findings demonstrate that heterozygous loss of *Shroom3* expression does not have any overt effects at the gross and histological level, except for a small proportion of hypoplastic unilateral kidneys seen in postnatal day 3- and 1-month old mice.

### Results 3: Shroom3^Gt/+^ Mice Demonstrate Altered Proximal Tubule Apical-Basolateral Orientation

**Figure 4.**
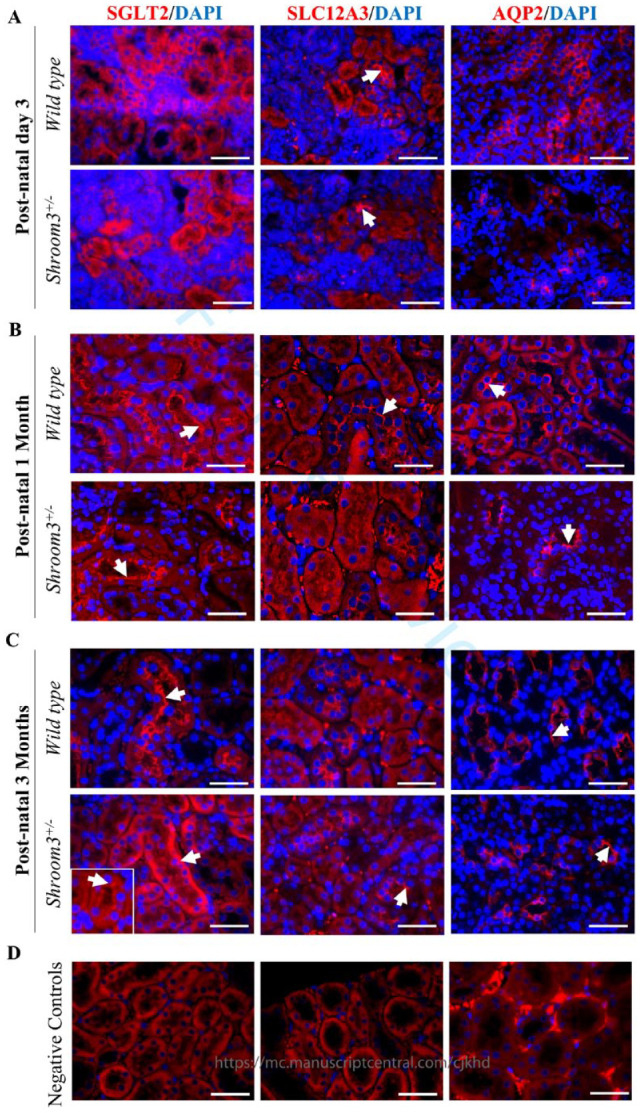
*Shroom3*^Gt/+^mice exhibit modest alterations in apical-basolateral orientation in 3-month-old mice. (**A-C**) Immunofluorescence of postnatal day 3, 1 month, and 3 months *wild type* and *Shroom3*^Gt/+^mice in the proximal convoluted tubules (SGLT2), distal convoluted tubules (SLC12A3), and collecting ducts (AQP2). (**A**) At postnatal day 3, SGLT2 and AQP2 show diffuse cytoplasmic expression. SLC12A3 shows strong apical localization in both *wild type* and *Shroom3*^Gt/+^mice. (**B**) At postnatal 1 month, all markers show strong apical localization in both *wild type* and *Shroom3*^Gt/+^mice. (**C**) At postnatal 3 months, all markers show strong apical localization in *wild type* mice. In *Shroom3*^Gt/+^ mice, SGLT2 localization is more consistent with cytoplasmic staining while SLC12A3 and AQP2 expression pattern is similar between *wild type* and *Shroom3*^Gt/+^mice. (**D**) Immunofluorescence staining of adult 3-month-old *wild type* kidneys demonstrate no specific background staining of the anti-rabbit secondary antibody in the absence of SLC12A3 as well as the anti-mouse secondary antibody in the absence of SGLT2 and AQP2. Scale bars = 25µm (A-D).

Our previous studies defined the effects of *Shroom3* null mutant mice on embryonic kidney development. In comparison to *wild type* mice, the *Shroom3* null mutant embryos demonstrated abnormal disrupted glomerular formation during earlier stages of kidney development (E13.5). The glomerular abnormalities observed at E13.5 were not obvious at E18.5, electron microscopy revealed abnormal podocyte morphology at later stages of kidney development (E18.5).^16^ Additionally, these studies observed a dose-dependent reduction in glomerular number in both *Shroom*3 homozygous, and to a lesser extent, heterozygous null mutant embryos in comparison to *wild type* at E18.5. This demonstrates that abnormalities during early kidney development can lead to disrupted glomerular formation and reduced glomerular number in later stages of development.^16^ In our present study, we extend these findings by investigating whether kidney developmental abnormalities in *Shroom3*^Gt/+^ embryos translate to abnormalities in the different nephron segments in postnatal and adult mice. Given that we did not observe any overt histological abnormalities in the glomeruli and renal tubular epithelia (**Figure 3**), we predicted that these nephron segment developmental abnormalities could manifest at the cellular and molecular level. As Shroom3 is primarily apically localized and regulates the apical actomyosin contractile networks, we examined if heterozygous deletion of *Shroom3* leads to altered epithelial apical-basolateral orientation in postnatal and adult kidney tissue.^8^ To test this, we compared immunofluorescence staining in *Shroom3*^Gt/+^ against *wild type* mice using the previously characterized apical markers (SGLT2- proximal convoluted tubule, SLC12A3- distal convoluted tubule, AQP2- collecting duct). At postnatal day 3 in both *wild type* and *Shroom3*^Gt/+^ kidneys (**Figure 4A**), SGLT2 and AQP2 showed a diffuse cytoplasmic expression pattern in several tubules, while SLC12A3 showed a strong apical localization in some tubules (**Figure 4A, arrows**). At 1 month (**Figure 4B**), both *wild type* and *Shroom3*^Gt/+^kidneys demonstrated a strong apical localization of all 3 markers (**Figure 4B**, **arrows**). However, at 3 months (**Figure 4C**), *wild type* kidneys showed a strong apical localization of SGLT2, whereas *Shroom3*^Gt/+^mice demonstrated an absence of apical SGLT2 expression in several, but not all, proximal convoluted tubules (**Figure 4C, arrow**). In some cases, the *Shroom3*^Gt/+^mice would show reductions in SGLT2 expression levels that maintain a normal apical expression pattern (**Figure 4C, arrow in inset)**. SLC12A3 also showed predominant apical localization at 3 months in *wild type* kidneys; however, in the *Shroom3*^Gt/+^ kidneys, SLC12A3 expression was mildly disorganized. Here, few cells demonstrated weaker apical SLC12A3 compared to *wild type* while some SLC12A3 expression was detected toward the basolateral aspect of distal tubular cells (**Figure 4C, arrows**). Lastly, AQP2 expression at 3 months demonstrated no marked differences between the *wild type* and *Shroom3*^Gt/+^ kidneys, although some *Shroom3*^Gt/+^ collecting ducts displayed broad apical staining (**Figure 4C, arrows**). Taken together, these results indicate that *Shroom3* heterozygous deletion results in age-dependent and segment-specific loss of apical integrity and abnormal tubular cell polarity that manifests by postnatal 3 months in the kidney cortex, predominantly in the proximal convoluted tubules.

### Results 4: Shroom3^Gt/+^ Mice Do Not Show Evidence of Proximal Tubular and Glomerular Injury

**Figure 5.**
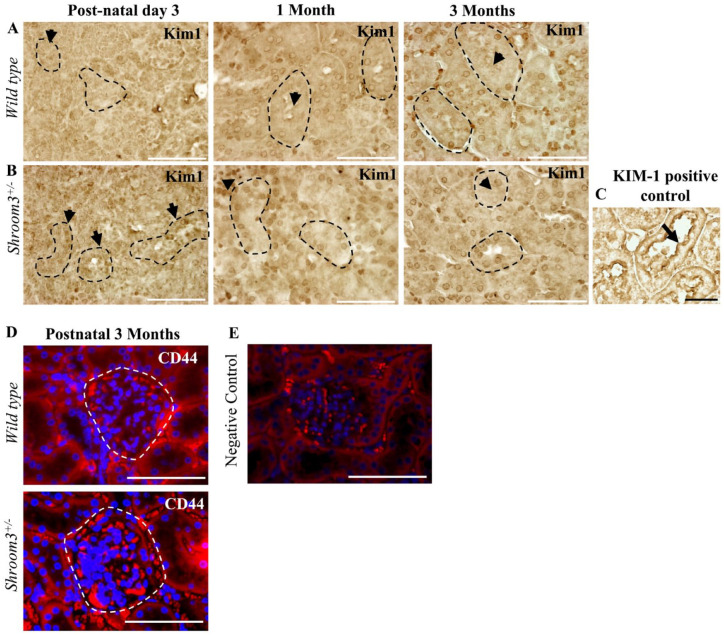
*Shroom3*^Gt/+^mice do not exhibit proximal tubular and glomerular injury. (**A**) Immunohistochemistry for KIM-1 at postnatal day 3, 1 month and 3 months in *wild type* mice demonstrating no significant KIM-1 protein expression. (**B**) Immunohistochemistry for KIM-1 at postnatal day 3, 1 month and 3 months in *Shroom3*^Gt/+^mice demonstrating no significant KIM-1 protein expression. (**C**) KIM-1 positive control for analysis. (**D**) Immunofluorescence staining of CD44 at postnatal 3 months in *wild type* and *Shroom3*^Gt/+^ mice demonstrate no significant differences in expression levels. *Shroom3*^Gt/+^ mice exhibit a thicker parietal epithelium than *wild type* mice. (**E**) Immunofluorescence staining of adult 3-month-old *wild type* kidneys demonstrate no specific background staining of the anti-mouse secondary antibody in the absence of the CD44 primary antibody. Scale bars = 50µm (A-E).

Previous studies have suggested that a loss of normal apical-basolateral orientation in renal epithelium can be associated with tubular injury.^24^ Given the disrupted apical integrity and altered epithelial cell polarity seen in the renal tubular epithelium (**Figure 4C**), we next sought to determine whether these abnormalities were accompanied by kidney injury using established biomarkers. First, we performed immunohistochemistry for Kidney Injury Molecule-1 (KIM-1), a widely used biomarker for renal proximal tubular injury (**Figure 5A-C**).^25^ In tissues with acute kidney injury, the damaged renal tubular epithelia exhibit KIM-1 staining on the apical surface of tubular cells (**Figure 5C**, KIM-1 positive [+ve] control from *wild type* mice after 48 hour ischemia reperfusion injury).^21^ This study revealed no significant levels of apical KIM-1 staining in either *wild type* or *Shroom3*^Gt/+^ kidneys at postnatal day 3, 1 month, and 3 months (**Figure 5A and B, arrows**). Next we performed immunofluorescence staining of CD44, a marker of activated parietal epithelial cells (PECs), which may contribute to glomerulosclerosis.^26-29^ The comparison between CD44 expression in *wild type* and *Shroom3*^Gt/+^ mice at 3 months, revealed no significant differences in expression levels (**Figure 5D**). However, we did notice that the *Shroom3*^Gt/+^ mice demonstrated a more prominent and thicker parietal epithelium consistent with an activated PEC phenotype (**Figure 5D, arrows**).^16,18^ Taken together, heterozygous *Shroom3* deletion was not significant enough to result in proximal tubular nor glomerular injury up to 3 months, yet our results demonstrated mild abnormalities in the parietal epithelium which may contribute to the development of glomerular pathologies and impaired kidney function overtime.

### Results 5: Shroom3^Gt/+^ Mice Do Not Show Renal and Cardiovascular Physiological Abnormalities

**Figure 6.**
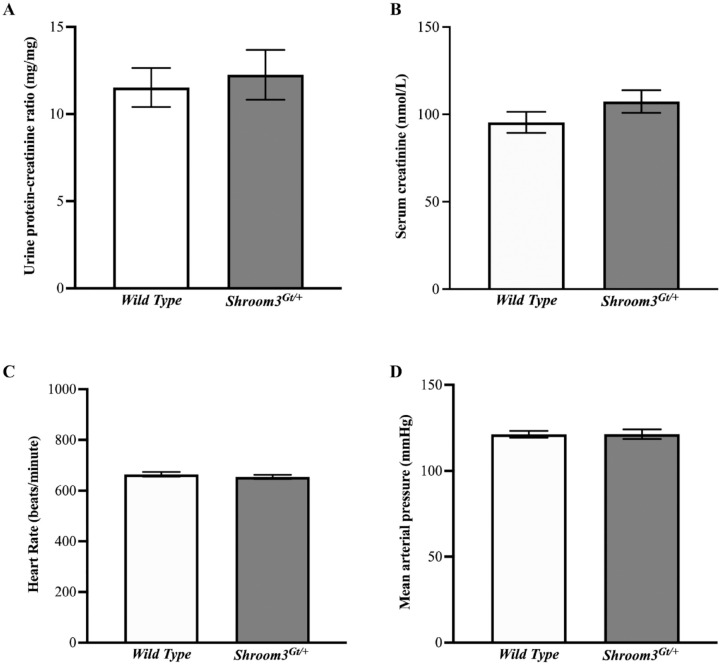
*Shroom3*^Gt/+^mice do not show renal or cardiovascular physiological abnormalities at postnatal 3 months. (**A**) Urine protein-creatinine ratio analysis in *wild type* and *Shroom3*^Gt/+^mice reveal slight increase in *Shroom3*^Gt/+^mice, however there is no statistically significant difference (*P* > .05). (**B**) Analysis of serum creatinine levels in *wild type* and *Shroom3*^Gt/+^mice reveal no statistically significant difference (*P* > .05). (**C**) Heart rate measurements are unchanged in *wild type* and *Shroom3*^Gt/+^mice. (**D**) Mean arterial blood pressure is unchanged in *wild type* and *Shroom3*^Gt/+^mice.

The most prominent molecular-level tubular abnormalities occurred in the proximal convoluted tubules of 3-month-old *Shroom3*^Gt/+^mice. This is made evident by the observed altered localization of the proximal convoluted tubule marker, SGLT2, in the *Shroom3*^Gt/+^kidneys (**Figure 4C**). As SGLT2 accounts for approximately 90% of glucose reabsorption from the glomerular filtrate, implications of its apical absence can include abnormal sodium-glucose plasma regulation which may relate to or even cause abnormalities in normal kidney and cardiovascular function.^30^ Therefore, we aimed to determine whether heterozygous loss of *Shroom3* has any physiological effects on the proper functioning of the renal and cardiovascular systems. To test this, we analyzed the protein/creatinine ratio, serum creatinine levels, heart rate, and blood pressure of postnatal 3-month-old male *wild type* and *Shroom3*^Gt/+^mice (**Figure 6**). The *Shroom3*^Gt/+^mice showed a slight increase in the urinary protein/creatinine ratio (**Figure 6A**) and serum creatinine levels (**Figure 6B**); however, these differences did not hold any statistical significance (*P* > .05). Additionally, there were no significant differences in heart rate (**Figure 6C**) or mean arterial blood pressure (**Figure 6D**) between *wild type* and *Shroom3*^Gt/+^ mice. Furthermore, we tested for the presence of glucose in urine and found no differences between the *wild type* and *Shroom3*^Gt/+^ mice. These findings demonstrate that heterozygous *Shroom3* deletion does not exert any detectable physiological abnormalities in renal and cardiac function in 3-month-old adult mice.

## Discussion

The data presented in this study provides evidence that *Shroom3*^Gt/+^ mice exhibit a mild kidney phenotype at 3 months, primarily at the molecular level. Consistent with the Shroom3 expression pattern, we detected altered apical-basolateral orientation of proximal tubular cells at postnatal 3 months as demonstrated by the reduced expression and lack of apical SGLT2 in the *Shroom3*^Gt/+^ kidneys. This mild kidney abnormality was not associated with changes in somatic growth, gross anatomy and histology, tubular morphology and integrity, or other cellular and molecular mechanisms in the *Shroom3*^Gt/+^mutants. However, we did observe hypoplastic unilateral kidneys in postnatal day 3- and 1-month old mice. Taken together, our results show that Shroom3 is important in maintaining apical integrity in the renal tubular epithelium; however, its heterozygous deletion is not a major contributing factor to abnormal kidney structure and function.

### Shroom3 Protein Expression in Adult Mice

Our previous studies characterized the expression pattern of Shroom3 in the developing kidney. Endogenous gene expression was examined through LacZ activity which reports the presence of Shroom3 expression. It was found that Shroom3 is strongly detected in the condensing mesenchyme located in the outermost part of the kidney cortex and in specific cells of the developing nephron structures including the developing and mature podocyte cell layer. Shroom3 was also detected in the ureteric bud stalks and tips of the developing collecting ducts.^16^ Our results expanded on the expression pattern and demonstrated Shroom3 expression in the proximal and distal convoluted tubules as well as the medullary collecting ducts. In all cases, expression was localized primarily to the apical regions of the cell. This Shroom3 expression pattern can translate to important functional roles in maintaining proper cell shape, providing tissue rigidity, and regulating the subcellular localization of various important molecules such as F-actin, RhoA, Rho-Kinases, and non-muscle-Myosin II.^6-8,17^ Additionally, Shroom3 may be responsible for apical cell-cell adhesion as it associates with the actin cytoskeleton in both adherens and tight junctions.^7,11,31,32^ Collectively, these studies demonstrate that the Shroom3 expression pattern is also detected in the postnatal period suggesting it has an important role in maintaining the tubular epithelium. This suggests a likely role for Shroom3 in cell communication, organization and regulation, and future studies could focus on uncovering the molecular significance of the Shroom3 expression pattern in the mature tubular epithelium cells of adult mice kidneys.

### Reduced Shroom3 Expression May Impair Branching Morphogenesis and Nephrogenesis

During kidney development, the ureteric bud undergoes branching morphogenesis and signals the nephron progenitor cells to form the various segments of the nephron. Bifurcations of the ureteric bud repeatedly occur throughout embryonic development to produce thousands of collecting tubules and millions of nephrons in order to establish the renal architecture.^33^ This highlights how interactions between the ureteric epithelium and nephron progenitor cells are required for kidney development. As previously described, Shroom3 demonstrated high expression levels in the early specialized mesenchyme progenitor cells with lower expression in the ureteric bud epithelium.^16^ Therefore, Shroom3 may play a role in kidney development where reduced expression may, in some instances, impair nephrogenesis and branching morphogenesis leading to an anatomically smaller kidney. This may explain the rare observation of the hypoplastic unilateral kidney seen to some degree in postnatal day 3 and more evidently in postnatal 1 month old *Shroom3*^Gt/+^ mice. Yet this observation was only seen in 3/14 of the 1-month-old *Shroom3*^Gt/+^ mice and these smaller kidneys did not exhibit any marked histological abnormalities. However, based on the expression pattern and function of Shroom3, we hypothesize that Shroom3 regulates the ability of the nephron progenitor cell population to aggregate and therefore its reduced expression may disrupt reciprocal signaling events in certain instances causing disturbances in normal kidney morphology. Further investigation is required to provide a better understanding of these processes in affected hypoplastic kidneys.

### Reduced Shroom3 Expression Alters Epithelial Polarity in Select Adult Nephron Segments

Shroom3 has an established role in generating the appropriate shape and function of epithelial tissues during embryonic development.^8,16,34^ It typically accomplishes cell shape changes and tissue morphogenesis through modulating the actin cytoskeleton at the apical surface of developing epithelial cells.^7,9,12^ Previous studies demonstrated this in the kidneys using embryonic *Shroom3* null mice where loss of apically distributed actin resulted in glomerular abnormalities including podocyte effacement and reduced podocyte numbers.^16^ Studies confirmed that reductions in Shroom3 expression postnatally causes defects in kidney function, such as albuminuria and podocyte foot process effacement.^16,18^ Our present study furthered the investigation on potential regulatory roles by looking at the relationship between Shroom3 and cell polarity in adult nephron segments. The SGLT2 immunofluorescence results demonstrated molecular abnormalities in the *Shroom3*^Gt/+^ kidneys that were primarily restricted to the proximal tubules; the nephron segment with the most prominent Shroom3 expression. Additionally, SLC12A3 and AQP2 did not demonstrate misexpression in the distal convoluted tubules nor the collecting ducts, respectively. Since recent evidence suggests that actin cytoskeleton dynamics may be involved in modulating ion channel localization, the disrupted apical integrity observed in *Shroom3*^Gt/+^ mice proximal tubules may be due to a lack of Shroom3 mediated actin organization and localization.^35-37^ However, more studies are needed to confirm if Shroom3 plays a role in actin-dependent ion channel regulation in the nephron. Altogether, our findings in combination with others demonstrate that decreased *Shroom3* levels in adult mice can lead to altered apical localization of key ion channels in the proximal tubule, possibly through a lack of Shroom3 mediated actin dynamics regulating proper ion channel localization.

### Reduced Shroom3 Expression May Impact Tubular Repair and Drive Secondary Glomerular Injury

In our present study, we found no evidence of overt histological damage at any of the studied ages. However, we found mild disturbances in the apical integrity of proximal convoluted tubules demonstrated by the marked apical absence and reduced expression pattern of the tubule marker SGLT2 at 3 months. Kidney function appeared normal as there were no detectable differences in urinary protein/creatinine ratios and serum creatine levels in 3-month-old *Shroom3*^Gt/+^ mutants. Our study did not detect any overt glomerular pathologies in postnatal *Shroom3*^Gt/+^ mice at 3 months, despite previous evidence of glomerular abnormalities during embryonic kidney development and glomerulopathy at 1 year.^16^ It is possible that the severe nephron alterations during development result in nephron death while the surviving nephrons exhibit changes at the molecular level. Therefore, the molecular changes found at 3 months may contribute to apical-basolateral disorientation which then prevents appropriate recovery from kidney injury. This concept was demonstrated by our recent publication that subjected *Shroom3*^Gt/+^mutants to ischemia reperfusion kidney injury. This study demonstrated that after acute kidney injury, *Shroom3*^Gt/+^mice were able to recover kidney function, yet exhibited worse kidney histopathology, specifically in the tubular epithelium.^21^ These findings could suggest a model where proximal convoluted tubular damage could drive secondary damage in renal corpuscles or even amplify existing damage. A similar study investigated sublethal diphtheria toxin-induced renal epithelial injury in proximal tubule cells and found that after an acute non-recurring injury, the tubules demonstrated a complete recovery. However, after repeated injury, the tubules were unable to repair properly resulting in glomerulosclerosis, demonstrating that glomerular damage occurred as a secondary effect of repeated acute proximal tubular injury. All in all, this study showed that long term repeated injury surpasses the self-repair capacity in the proximal tubules leading to tubular damage and inflammation that may then drive postnatal glomerular damage.^38^ Comparably, our present findings demonstrated that Shroom3 deficiency initially results in mild damage in the proximal tubules and does not immediately affect the glomeruli or other segments of the nephron. However, over the period of 1 year, the cellular damage in the proximal tubules progressively worsened, causing secondary damage to the renal corpuscles.^16^ Our findings suggest that the observed mild molecular abnormalities seen in *Shroom3* heterozygous null mice at 3 months may not immediately contribute to abnormal kidney structure and function, however abnormalities may manifest with age or in the event of external insults. Taken together, the abnormal molecular phenotype seen with reduced *Shroom3* expression at 3 months may contribute to increased sensitivity to kidney disease and worsened tubular repair and glomerular disease after a kidney insult or with aging.

## Conclusion

In conclusion, the heterozygous loss of *Shroom3* in mice does not result in overt kidney damage or decreased kidney function. Rather, the only mild abnormality detected in the *Shroom3*^Gt/+^mutants was the altered apical orientation of proximal tubular cells which did not translate to tubular damage or decreased kidney function. These findings set the important groundwork establishing that *Shroom3* genetic mutations alone do not cause kidney disease. Yet, there is a possibility that individuals with *Shroom3* genetic anomalies may experience worsened kidney disease when in the presence of an external insult, such as acute kidney injury, an area that requires further exploration in future studies.
